# Promising Therapeutic Strategies Against Microbial Biofilm Challenges

**DOI:** 10.3389/fcimb.2020.00359

**Published:** 2020-07-28

**Authors:** Kaiyu Zhang, Xin Li, Chen Yu, Yang Wang

**Affiliations:** ^1^Department of Infectious Diseases, First Hospital of Jilin University, Changchun, China; ^2^Department of Pediatrics, University of Oklahoma Health Sciences Center, Oklahoma City, OK, United States

**Keywords:** biofilm, antibiotic resistance, antimicrobial peptides, nanotechnology, combination therapies

## Abstract

Biofilms are communities of microorganisms that are attached to a biological or abiotic surface and are surrounded by a self-produced extracellular matrix. Cells within a biofilm have intrinsic characteristics that are different from those of planktonic cells. Biofilm resistance to antimicrobial agents has drawn increasing attention. It is well-known that medical device- and tissue-associated biofilms may be the leading cause for the failure of antibiotic treatments and can cause many chronic infections. The eradication of biofilms is very challenging. Many researchers are working to address biofilm-related infections, and some novel strategies have been developed and identified as being effective and promising. Nevertheless, more preclinical studies and well-designed multicenter clinical trials are critically needed to evaluate the prospects of these strategies. Here, we review information about the mechanisms underlying the drug resistance of biofilms and discuss recent progress in alternative therapies and promising strategies against microbial biofilms. We also summarize the strengths and weaknesses of these strategies in detail.

## Introduction

**Figure 1:**
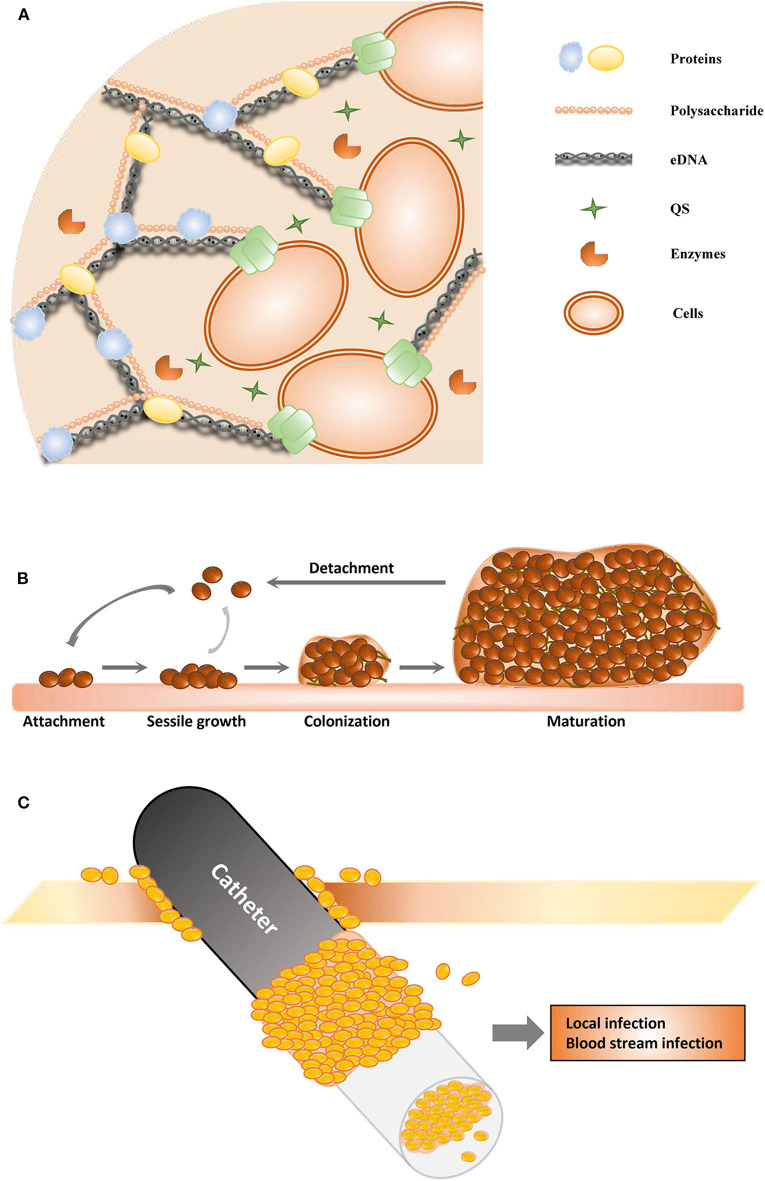
Schematic of biofilm formation. **(A)** The structure of the extracellular polymeric substance. **(B)** The stages of biofilms. **(C)** The catheter-associated biofilm.

Most microorganisms develop several types of survival mechanisms to adapt to surrounding conditions and to sustain activity against host immune responses and antimicrobial treatment. Biofilms are groups of microorganisms attached to biotic or abiotic surfaces and surrounded by a matrix composed of an extracellular polymeric substance (EPS) (Figure 1A) (Fulaz et al., 2019). The metabolic activity, genetic adaptation, and communication of the microorganisms within microbial biofilm communities are altered (Singh et al., 2017). Biofilms exist in various infections and have been demonstrated to play an important role in human diseases. Biofilms act as physical barriers against drugs and host immune responses, leading to resistance to antimicrobial treatment. Biofilms obviously reduce the possibility of eradicating infections and cause relapses after the traditional appropriate treatment. The onset of biofilm-related infections can increase not only severe symptoms but also mortality (Tascini et al., 2018). Given that more studies are focusing on strategies to eliminate microbial biofilms, it is time to better understand the roles of biofilms in infections in depth and to carefully assess the latest promising antibiofilm strategies reported in the published literature. Here, we review information about the mechanisms of biofilm drug resistance and recent progress in alternative therapies and promising strategies against microbial biofilms. We also summarize the strengths and weaknesses of these strategies in detail.

## Biofilms and their Roles in Infections

### Biofilm Formation

Biofilms are important virulence factors of some pathogenic microorganisms, and some biofilm infections seem nearly impossible to eradicate (Zarnowski et al., 2014). Most bacteria and fungi, such as *Pseudomonas aeruginosa* (Olivares et al., 2020), *Staphylococcus epidermidis* (Sabaté Brescó et al., 2017), *Candida albicans* (Tsui et al., 2016), *Acinetobacter baumannii* (Eze et al., 2018), *Helicobacter pylori* (Yonezawa et al., 2015), *Staphylococcus aureus* (Moormeier and Bayles, 2017), *Listeria monocytogenes* (Barbosa et al., 2013), *Vibrio cholerae* (Bridges and Bassler, 2019), and *Salmonella enterica* (Fàbrega et al., 2014), can form biofilms. All these microorganisms form biofilms in a similar manner and share many common features (Koo et al., 2017; Moormeier and Bayles, 2017; Cavalheiro and Teixeira, 2018). Biofilms are one of the most important health threats, causing nearly 80% of refractory nosocomial infections (Jamal et al., 2018). Biofilm-related infections can be divided into medical device- and tissue-associated biofilm infections (Römling et al., 2014). The details of these two types of biofilm-related infections are discussed in the next section. Most pathogenic microorganisms are associated with infections related to medical devices, such as urinary catheters, orthodental prosthetics, pacemakers, cardiovascular valves, contact lenses, and breast implants (Percival et al., 2015b). The process of biofilm maturation is complex and sometimes distinctive for survival in various harsh environments (O'Toole et al., 2000). Commonly, mature biofilms can be regarded as populations or communities of microorganisms attached to a range of biotic or abiotic surfaces. Biofilms can comprise a single or multiple microbial species. Abiotic surfaces can be food surfaces and surfaces within homes and public places, and these abiotic surfaces can become infectious reservoirs (Lappin-Scott and Bass, 2001). Microorganisms initiate biofilm formation due to specific environmental pressure, such as nutrition or antibiotic treatment. The cells attach themselves to surfaces via hydrophobic interactions or by binding to surface proteins in a particular manner, such as to host matrix proteins that surround catheters or implants. Biofilm formation mainly involves three stages (Figure 1B). During the first stage (adhesion stage), cells attach to a surface; in the second stage (sessile growth stage), an assembly of these cells forms microcolonies. The adhesion and sessile growth stages are reversible, and the cells can cluster loosely but can detach and return to a planktonic state (Kumar et al., 2017). Then, the attached cells secrete EPS, which includes extracellular DNA (eDNA), proteins, and polysaccharides (Figure 1A), and develop to form a biofilm in the third stage. This stage is irreversible. In the third stage of biofilm formation, the cells are attached within a thick and stable complex biomolecular layer (Roy et al., 2018). The fully matured biofilm looks like a three-dimensional tower structure and provides shelter for the cells within. After a biofilm is completely developed, its dispersion or disassembly occurs via both active and mechanical processes. These processes occur in the fourth stage (dispersal stage). The cells within the biofilm secrete not only cell–cell-adhesive matrix components but also disruptive factors, including phenol-soluble modulins, proteases, nucleases, and regulators (Graf et al., 2019). These disruptive factors can also promote biofilm detachment. During the process of detachment, biofilms can shed individual cells and slough off pieces into the bloodstream and the surrounding tissues, which is associated with many acute and chronic infections (Davies, 2003).

The organization of microorganisms in biofilms endows the component cells with some properties that are not distinct in individual cells that are independently grown or that are in planktonic populations in liquid media. In addition, many of these activities are associated with the formation of mixed-species functional groups within the EPS, while others are associated with the formation of physicochemical gradients in the biofilm that regulate the metabolism of the component cells. The biofilm is the most stable state within the biological cycle among planktonic cells, cells attached to the surface, and mature biofilms, especially in challenging situations. These biofilms continue to grow when nutrients are plentiful; however, the cells within the biofilm transition to a planktonic mode as nutrients are depleted. Different microbial biofilms exhibit similar stages of biofilm development and show similar roles as a protective cover, e.g., *P. aeruginosa* (Olivares et al., 2020), *S. epidermidis* (Sabaté Brescó et al., 2017), and *C. albicans* (Tsui et al., 2016) biofilms. The synthetic and metabolic characteristics of microorganisms can change when the organisms switch between the free-living and biofilm growth modes (Bell, 2001). Within biofilms, cells with different phenotypes and genotypes co-express individual metabolic pathways, stress responses, and other distinct biological properties. Some cells alter extracellular polysaccharide and organelle production and even cell morphology after they sense growth within the biofilm community. The populations within biofilms are complex and exhibit chemical, physiological, and genetic heterogeneity (Stewart and Franklin, 2008). Although most studies focus on biofilms formed by a single microorganism, multiple microbial species can exist within a biofilm, such as in oral multispecies biofilms (Kolenbrander et al., 2010). DNA transfer and genetic recombination between the multiple microbial species within a biofilm occur without direct cell–cell contact through the extracellular matrix. Antibiotic resistance genes can be transferred in this manner (Kolenbrander et al., 2010). Quorum sensing (QS) is a cell–cell communication process that regulates multiple physiological and biochemical functions. An early study demonstrated that QS is involved in regulating the formation of a *P. aeruginosa* biofilms (Davies et al., 1998). Structurally, QS signal molecules have a low molecular weight and belong to a wide range of chemical classes, including acyl homoserine lactones (AHLs), autoinducer peptides, autoinducer-2, and cis-unsaturated fatty acids (DSF family signals). The production of AHLs was first observed in biofilms in 1998 (Stickler et al., 1998), and 1 year later, several studies on *P. aeruginosa* biofilms identified that QS was involved in biofilm development (Hassett et al., 1999). Later, it was found that QS also played important roles in Gram-positive bacterial biofilm formation (Wuc et al., 2019). It is now well-known that QS controls and facilitates biofilm formation in many bacterial and fungal species, causing the production of antibiotic resistance and virulence factors (Madhani, 2011; Hong et al., 2012). It has been widely acknowledged that QS is necessary for genetic-level regulation and population-level dynamics and plays vital roles in biofilm development. Microorganisms use QS to regulate the population density to optimize metabolic production (Wuc et al., 2019). QS regulates not only the maturation but also the disassembly of the biofilm community by inhibiting the synthesis of matrix compounds or the degradation of the matrix in a coordinated manner (Solano et al., 2014). According to the important effects of QS on biofilm formation and development, numerous studies have tried to inhibit biofilms by targeting the production of these autoinducers or by blocking their receptors. QS inhibitors are regarded as promising antibiofilm agents (Whiteley et al., 2017).

### Medical Device-Associated Biofilm Infection

With medical improvements, medical devices (such as contact lenses, orthodontal prosthetics, endotracheal tubes, central venous catheters, needleless connectors, intrauterine devices, cardiovascular valves, pacemakers, peritoneal dialysis catheters, urinary catheters, prosthetic joints, and breast implants) are widely used and have become essential for treatments in clinical work. Sometimes, the use of medical devices is associated with complications as well, and the most common secondary complication is infection due to microorganisms that detach from biofilms on the medical device; an example of this is catheter-associated biofilms (Figure 1C) (Donlan, 2001). Urinary catheter-associated biofilms were observed in 1985, and antibiotic resistance of the biofilm was reported (Nickel et al., 1985). Catheter-associated urinary tract infections are very common, and many studies have focused on these infections. Microorganisms in biofilms on the inner surface of catheters in patients with long-term catheterization are protected from antibiotic treatment and cause chronic infection (Delcaru et al., 2016). It has been indicated that biofilm formation on urinary catheters occurs mainly by one of two routes. Microorganisms may colonize the outside surface of the catheter by direct inoculation during catheterization or by migration through the surrounding mucous sheath. Most related microorganisms come from the gastrointestinal tract, colonizing the perineum (Delcaru et al., 2016). Most urinary catheter-associated biofilms occur through extraluminal entry of microorganisms, especially in female patients (Delcaru et al., 2016). Microorganisms can enter the catheter by an intraluminal route and form a biofilm due to a failure to maintain a closed drainage system or when a collection bag is contaminated (Nickel and Costerton, 1992). Microorganisms can also enter the urinary tract and form a catheter-associated biofilm through a bloodstream infection; however, it is more common that a urinary tract infection is the main cause of sepsis.

In later studies, other catheter-associated biofilms and implanted material-associated biofilms were studied widely, including biofilms associated with contact lenses, orthodontal prosthetics, endotracheal tubes, needleless connectors, central venous catheters, intrauterine devices, cardiovascular valves, pacemakers, prosthetic joints, and breast implants (Zahran et al., 2015; Sampaio et al., 2016; Gominet et al., 2017; Okuda et al., 2018; Stewart and Bjarnsholt, 2020; Walker et al., 2020). The presence of a biofilm is thought to accompany infection or colonization of chronic peritoneal dialysis catheters and to be an important pathogenetic factor in the recurrence or persistence of peritonitis (Sampaio et al., 2016). Catheter-associated bloodstream infection is an important cause of nosocomial infections with significant associated morbidity (Bouza et al., 2015). Biofilm formation on a catheter may originate from contaminating microorganisms during surgery and/or catheter insertion. These biofilms form on the outside surface of the catheter. *S. epidermidis, S. aureus*, and *C. albicans* are common microbes on the skin; therefore, they are the most important pathogenic cause of catheter-related biofilm infections (Septimus and Schweizer, 2016). In addition, biofilms in the catheter lumen can originate from bacteremia. Compared to planktonic cells, cells within biofilms produce fewer proinflammatory factors, which normally cause considerable host responses. Many microorganisms can colonize and form biofilms in endotracheal tubes. Biofilms in endotracheal tubes are related to ventilator-associated pneumonia, one of the most common infections and leading causes of death in intensive care units (Orhan-Sungur and Akça, 2006; Fernández-Barat and Torres, 2016). Increasing evidence indicates that biofilm formation on long-term medical implants, such as prosthetic joints, pacemakers, heart valves, contact lenses, and breast implants, leads to major postoperative complications. Infections can cause inflammation and tissue destruction around implants, and sometimes, these infections are life threatening. Because of the difficulties in eliminating biofilms, implant replacement needs to be considered in many patients (Arciola et al., 2018).

Medical-device-associated biofilms are the most important sources of nosocomial infections. Most studies on biofilms carried out among important opportunistic pathogens have been comprehensive. Biofilms are most commonly formed by *S. epidermidis* (Sabaté Brescó et al., 2017) and *S. aureus* (Moormeier and Bayles, 2017), while other nosocomial opportunistic microorganisms, such as *P. aeruginosa* (Nickel et al., 1985; Hassett et al., 1999; Bell, 2001), *Escherichia coli* (Koseoglu et al., 2006), *Klebsiella pneumoniae* (Stahlhut et al., 2012), *A. baumannii* (Eze et al., 2018), and *C. albicans* (Chandra et al., 2001), can also form medical-device-associated biofilms. Most of these pathogens are multidrug resistant, and the treatment of these biofilms is very challenging.

### Tissue-Associated Biofilm Infection

Microorganisms may also adhere to biotic surfaces and form biofilms in different tissues in the host, e.g., epidermal cells (Paranjpye and Strom, 2005) and teeth (Black et al., 2004), or they may be located in tissues, e.g., in the mucus on mucosal membranes (Cellini et al., 2008) or inside chronic wounds (Akiyama et al., 1996). Biofilms formed in gingival crevices and on tooth surfaces are regarded as the major causes of the pathogenesis of gingivitis and periodontitis, and they may be related to the synergistic effect of polymicrobes and dysbiosis. An increased risk of cancer is associated with persistent inflammation and chronic infections (Groeger and Meyle, 2019). Up to 750 types of microorganisms, including viruses, protozoa, archaea, fungi, and bacteria, have been recognized in the oral microbiome. Oral biofilm formation by multiple species, such as *Streptococcus* and *Actinomyces*, is very common. Tooth surface biofilms can lead to dental caries, and periodontal disorders can be induced by supra- and subgingival biofilms below and along the gingival area (Mosaddad et al., 2019). It has been identified that multiple gastrointestinal infections can be caused by biofilm formation. Biofilm formation on human gastric mucosa by *Helicobacter pylori* has been observed in endoscopically directed biopsies with scanning electron microscopy (Carron et al., 2006). It is difficult to eradicate an *H. pylori* infection because of biofilm formation. *Salmonella* can form biofilms on human gallstones, and bile can significantly enhance the biofilm formation of *Salmonella*. The biofilm of *Salmonella* on gallstones may be a source of chronic infection and is related to a high risk for developing gallbladder cancer (Prouty et al., 2002). Multiple species of microorganisms, such as *E. coli* (Conway and Cohen, 2015), *V. cholerae* (Silva and Benitez, 2016), and *S. enterica* (Azriel et al., 2015), can form biofilms in host intestines. The normal flora of female genitalia includes both avidly and loosely tissue-adherent bacterial biofilm populations (Davies, 2003). Probiotics, which are live bacteria and yeasts used in the treatment and prevention of diarrheal diseases and help keep the gut healthy, can also form biofilms (SlíŽová et al., 2015). On the other hand, it has been shown that efficient biofilm formation of commensal/probiotic-type strains can confer an advantage, protecting the host against pathogens and reducing the incidence and severity of enterocolitis (Olson et al., 2016).

## Mechanisms of Biofilm Resistance to Antimicrobial Agents

The biofilm growth mode offers protection against many biocides and antibiotics; thus, biofilms are hard to control and ultimately eradicate. It has been indicated that microorganisms resuspended from biofilms are distinctly more resistant than planktonic cells, while the cells inside biofilms are more resistant than those resuspended from biofilms. Biofilm cells are at least hundreds of times more resistant to antibacterial agents than planktonic cells (up to 1,000-fold increase) (Roy et al., 2018). Biofilms protect cells from desiccation, chemical perturbation, invasion by other bacteria, and killing by immune cells by acting as shelters or physical barriers (Yan and Bassler, 2019).

**Figure 2:**
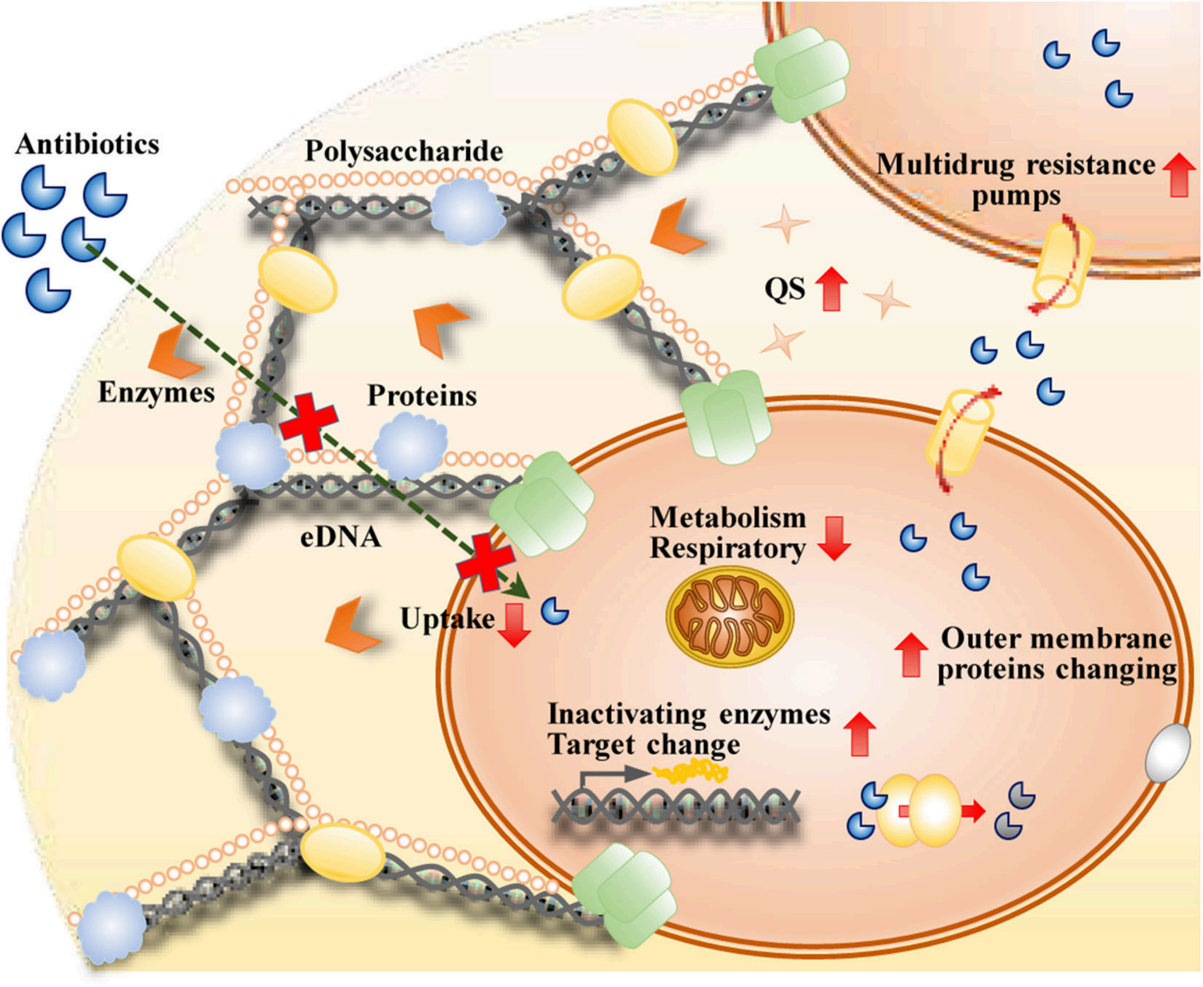
Mechanisms of biofilms that are resistant to antibiotics therapy.

There are multiple mechanisms by which biofilm cells create increased resistance to antibiotics, and these mechanisms are distinct from those in planktonic cells (Figure 2). Impeded antibiotic penetration into biofilms was initially proposed to be responsible (Dunne, 1993). However, penetration by some antimicrobial agents, such as ciprofloxacin and fluconazole, throughout biofilms does not decrease (Anderl et al., 2000). It is now well known that the matrix mesh size is much larger than the size of antibiotic molecules (Yan et al., 2018). The penetration of antimicrobials into a biofilm depends on the thickness of the biofilm, the reactivity and diffusion of the agent within the biofilm, the sorption of the biofilm, and the dose concentration of the agent (Stewart, 2015). The production of an exopolysaccharide matrix reduces the activity of some antibiotics, such as fluconazole, in *C. albicans* biofilms (Nett et al., 2007). eDNA can reduce antibiotic activity by creating cation-limited conditions, inducing modification of lipopolysaccharide (LPS), and impairing the uptake of antibiotics, such as aminoglycosides (Mulcahy et al., 2008). eDNA is regarded as one of the most important contributors to the resistance of biofilms to antimicrobial agents.

It is widely accepted that the status of the cells within biofilms is associated with their sensitivity to antimicrobials. The higher osmolarity conditions, greater oxygen limitations, higher metal ion concentrations, and lower pH levels within a biofilm are confirmed to be responsible for the expression of some genes and help determine some of the phenotypes of biofilm cells (Prigent-Combaret et al., 1999). Within a biofilm, the concentration of oxygen is higher at the surface and lower at the bottom and the center (Costerton et al., 1995). Accordingly, cells with a high level of metabolic activity are located at the surface of the biofilm, and those with a low level of metabolic activity and slow growth are present in the center. Metabolically active cells are able to sense environmental changes and actively respond to the presence of antimicrobial stress. However, a majority of the cells inside biofilms are in a dormant state and in the stationary phase, which means that these cells are metabolically inactive and not growing. Cells in the stationary phase within a biofilm do not grow and respire and are more tolerant to antimicrobials (Stewart, 2015). Changed nutrient environments and inhibition of growth within the biofilm lead to increased drug resistance within biofilms.

Intrinsic mechanisms of resistance are present in biofilms, but many studies have indicated that the synergy of acquired and adaptive mechanisms contributes to antibiotic resistance in biofilms (Taylor et al., 2014). Genetic adaptation within biofilms helps cells adapt to their surroundings and increases their antibiotic resistance. Changes in the outer membrane proteins of the cells within biofilms contribute to antibiotic resistance via the expression of multidrug resistance genes. Some antibiotics can induce resistance-related enzyme expression in the cells within biofilms. For example, high-level imipenem resistance is related to increased beta-lactamase expression induced by imipenem in *P. aeruginosa* biofilms. Piperacillin can also induce beta-lactamase expression in biofilms; however, the increased beta-lactamase expression is not as high as the imipenem level. The combination of increased beta-lactamase expression with other protective properties of the biofilm growth mode is the main reason for biofilm persistence in chronic infections (Coquet et al., 1998). Changes in the activities of multidrug efflux pumps in biofilms contribute to drug resistance. The activated efflux pumps of the cells within biofilms have received the most attention (Kean et al., 2018). Persister cells, the prevalent cell population in the stationary state of biofilm communities, are dormant cells in the microbial subpopulation and are phenotypic multidrug-tolerant variants rather than genetic ones (Keren et al., 2011). The other mechanism of antibiotic resistance of biofilm cells is the acquisition of multidrug resistance genes by horizontal transfer, which contributes to the evolution of the cells within biofilms (Mah, 2012). QS has an essential effect on this horizontal transfer between the cells inside biofilms (Zhu et al., 2019). Evidence suggests that biofilms have developed these mechanisms as a general stress response that induces the microorganisms in the biofilm to react to these environmental changes that they may experience (Mah, 2012). To fight biofilms, novel strategies targeting these mechanisms need to be developed.

## Strategies Against Microbial Biofilms

**Figure 3:**
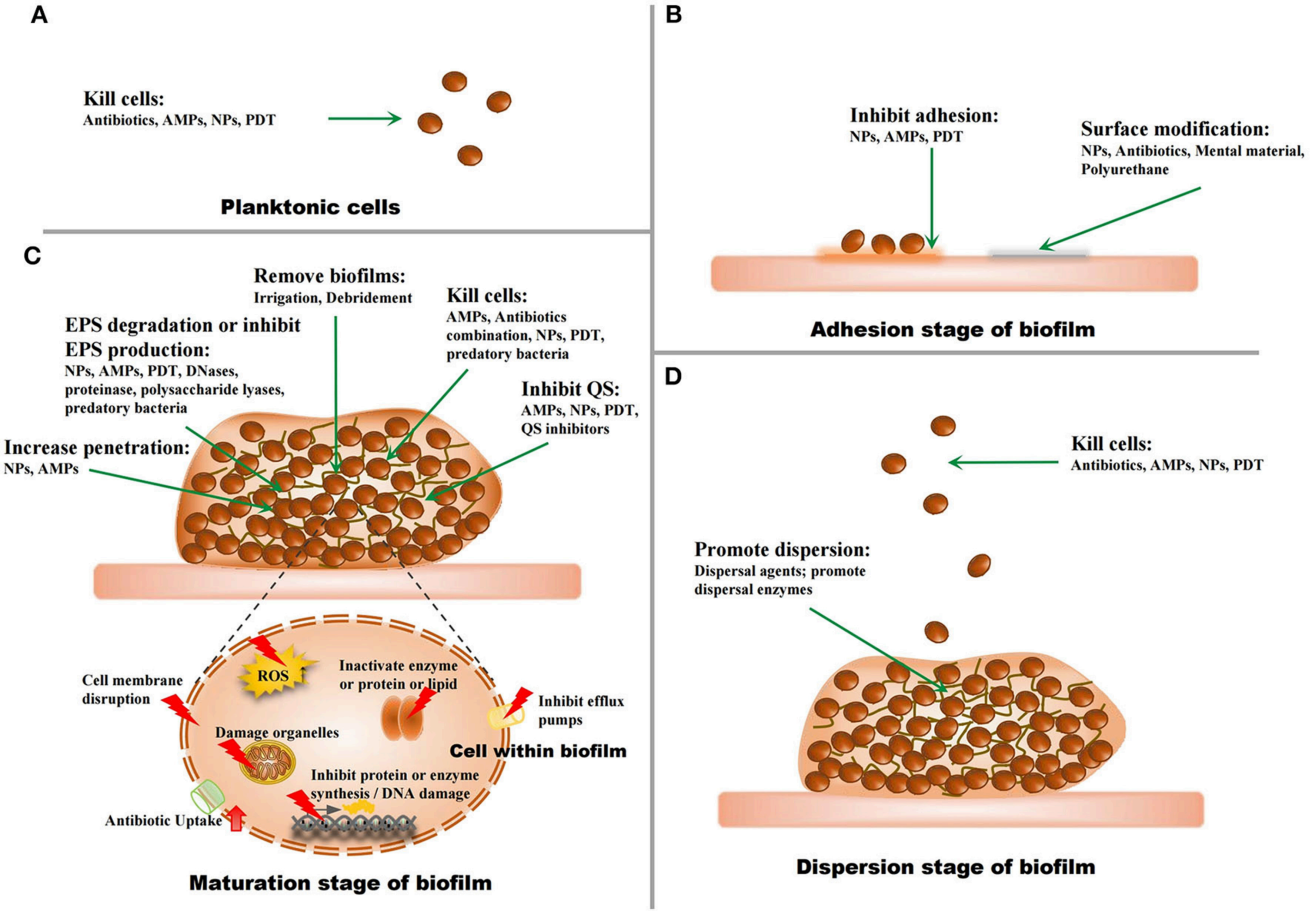
The strategies against biofilms. **(A)** The strategies for planktonic microorganisms. **(B)** The strategies for the adhesion stage of biofilms. **(C)** The strategies for the maturation stage of biofilms. **(D)** The strategies for the dispersion stage of biofilms.

Since biofilm-related infections and the challenges in their treatment have been regarded as major threats to human health, strategies to address this problem have been developed in recent years (Figure 3).

### Traditional Antibiotic Combination Treatments

Cells inside biofilms have a much higher minimum inhibitory concentration (MIC) of antibiotics, and high antibiotic concentrations can be provided through topical administration (Olivares et al., 2020). There are a limited number of new antibiotics and therapeutic options against biofilms, necessitating the development of new strategies to find novel antibiofilm candidates. Because of the resistance of biofilms to antibiotic treatments, combination therapy with different medicines was considered to try to eradicate biofilms. In an initial attempt, an *E. coli* biofilm was treated with a combination of the antibiotics amdinocillin and cefamandole in 1987 (Prosser et al., 1987). Researchers continue to try various combination schemes to eliminate biofilms through synergistic effects and are also trying sequential/alternate therapies and high-dose topical treatments (Akturk et al., 2019). Most studies have focused on common multidrug-resistant pathogens that cause nosocomial infections, such as *E. coli, P. aeruginosa, S. aureus*, coagulase negative staphylococci, and *C. albicans* (Cai et al., 2009; Pettit et al., 2009; Sarkar et al., 2014; Wang Y. et al., 2014; Ahmed et al., 2019). Many studies have identified the synergistic activities of traditional antibiotics not only *in vitro* but also *in vivo* (Cai et al., 2009; Pettit et al., 2009). Multiple factors need to be considered, such as the susceptibility, targets of the antibiotics, permeability, and PK/PD in the biofilm of the antibiotics, when a combination therapy is used (Cernohorsk and Votava, 2008; Dales et al., 2009; Rose and Poppens, 2009). Many combination antibiotic therapy schemes have been used in clinical case studies (Dales et al., 2009). The synergism of antibiotics and other kinds of medicine has also been identified, such as that of sodium salicylate and *N*-acetylcysteine (Polonio et al., 2001; Belfield et al., 2017). These medicines include anti-inflammatory reagents, immunomodulatory reagents, and drugs used to break up the extracellular matrix and eradicate biofilms (Bernal-Mercado et al., 2020). However, biofilms can be difficult to thoroughly remove because the dose of antibiotics is limited by their side effects (Ciofu et al., 2017). Consequently, considerable attention has been paid to new agents and technological developments, although the most promising alternative still needs to be based on the combination of new agents with traditional antibiotics.

### Irrigation and Debridement to Physically Remove Biofilms

Irrigation with a dental water jet was reported to be effective in removing biofilms both *in vivo* and *in vitro* (Gorur et al., 2009; Kato et al., 2012). Proper antimicrobial skin preparation can reduce bacterial populations and wound biofilm formation (Paulson, 2005). For oral, wound, and prosthetic joint biofilm-related infections, irrigation with water jets and debridement followed by aggressive antimicrobial therapy has been widely carried out, although it was also found that biofilms spread across the surface and cause the persistence of bacteria on surfaces after these treatments (Urish et al., 2014; Fabbri et al., 2016). The treatment of prosthetic joint biofilm-related infections is regarded as complex, and the choices are different and distinct according to the pathogen and the duration of infection onset (Aboltins et al., 2014). Some proper detergents, such as a chlorhexidine gluconate scrub, are reported to be helpful in decreasing colony counts and removing biofilms (Schwechter et al., 2011). Some new technologies, such as low-intensity intermittent ultrasonication-induced bursting of microbubbles (Agarwal et al., 2014) and cavitating jets (Yamada et al., 2017), have been combined with these traditional therapies. While these novel methods are promising, their efficacy has yet to be shown in the clinic.

### Surface Modification

The threat of medical-device-associated biofilm infection has increased. The attachment of microorganisms to a surface is a critical step in biofilm development, and once biofilms develop on a medical surface, the eradication of biofilms becomes very difficult. Therefore, many studies have focused on modifying the surfaces of medical devices as a major strategy to eliminate biofilms. To combat biofilm formation, coatings for medical prostheses have been widely developed. Metal materials have been found to have anti-infective efficacy and have also been used to coat catheters. Silver or silver–copper multilayer coatings used in various catheters and other medical devices, including urinary catheters, peritoneal catheters, vascular catheters, and fracture fixation devices, prevent the growth of biofilms (Bechert et al., 1999). Current preventive approaches for surface modification work mainly through physical pretreatment or coating of the surfaces with antimicrobial agents or agents that reduce adhesion. In 1995, central venous catheters coated with silver sulfadiazine and chlorhexidine were implanted in swine and indicated to have non-toxic ranges (Greenfeld et al., 1995). Then, rifampicin, minocycline, and gentamicin were used in studies on biofilm surface modifications (Spencer, 1999; Cho et al., 2001). Commercial coated catheters are coated with broad-spectrum antibiotics, such as chlorhexidine, minocycline, rifampin, and silver sulfadiazine, and these catheters have been used widely in clinical studies, especially in intensive care units, according to the recommendations supported by some guidelines (Dwyer, 2008). It has been demonstrated that antimicrobial catheters improve outcomes even in the presence of bacteremia (Jamal et al., 2014). Hydrogels have been used to coat medical devices and have been shown to be effective in combating biofilms because of their good functional group density, biocompatibility, and lubricity (Norris et al., 2005). Polyurethane was developed as a biodegradable polymer and could deliver controlled doses of antibiotics. Antifouling polyurethanes have been estimated to have antibiofilm activity and may be utilized as coating materials for medical implants (Tunney and Gorman, 2002). Nanotechnology has been used for surface modification to fight against biofilms, and detailed information about this new technology will be discussed in the next section.

### Nanotechnology

Nanotechnology is a well-established scientific and engineering technology. Nanomaterials (NMs) have at least one dimension in the nanometer-scale range of 1–100 nm and have some special physical and chemical properties. NMs have been developed in diverse medical diagnostic and therapeutic fields. Various NMs, such as lipid (Rout et al., 2017), polymer (Landis et al., 2017), and metal NM (Besinis et al., 2014), have been produced. Metal NMs have become the core materials because of their non-toxic nature and essential inertness (Burygin et al., 2009). Nanotechnology can play various roles in combating biofilms, not only by directly killing or inhibiting microbes but also by carrying antibiotics or other agents with antibiofilm activity (Li et al., 2019). NMs can also be utilized as delivery carriers due to their small size. With nanotools, traditional antibiotics and other novel antimicrobial agents can pass through the biofilm barrier and penetrate further into the biofilm, killing the cells inside the biofilm (Galdiero et al., 2019). The main nanocarrier types include molecular complexes (such as protein nanocomplexes and cyclodextrin nanocomplexes), polymer-based nanocapsules [such as dendrimers, core–shell nanocapsules, and ligand-decorated nanoparticles (NPs)], inorganic nanocarriers (such as metal NPs), and lipid-based nanovesicles (such as liposomes and solid lipid NPs). Among the many kinds of NMs, NPs have attracted particular attention (Sambhy et al., 2006). As a carrier, NPs can enhance the solubility and stability of drugs (Ding et al., 2018) and increase the biocompatibility of drugs at the target site (Burygin et al., 2009). The release of the delivered drug can be controlled by different stimuli, such as the salt concentration, pH, light, and temperature (Paasonen et al., 2007).

These new NPs, especially metallic NPs, enhance the antimicrobial effect of existing antibiotics and present their own bactericidal activity. Metallic NPs can release metal ions that interact with cellular components through various pathways to fight biofilms (Paasonen et al., 2007). The major antibiofilm mechanisms of NPs are as follows. (1) NPs can come in direct contact with the microbial cell wall and damage the cell wall and cell membranes. This antimicrobial effect of NPs has been identified in Gram-negative and Gram-positive bacteria and fungi (Grigor'eva et al., 2013; Monteiro et al., 2013). (2) NPs can prevent the surface adherence of microorganisms and inhibit biofilm formation. NPs are a promising technology to eradicate or inhibit biofilms and increase the transport of antimicrobials to the neighborhood of the cell. Alternatively, NPs could carry matrix dispersion agents. The penetration within the biofilm is mostly controlled by the size of the NPs (Habash et al., 2014; Ding et al., 2018). (3) NPs can regulate host immune responses to inhibit pathogenic biofilm formation, and NPs have anti-inflammatory properties (Shi et al., 2019), although it has also been shown that some NPs, such as zinc oxide NPs (Lin et al., 2014), impair innate immune responses. (4) NPs, such as silver NPs (Paosen et al., 2019), can generate reactive oxygen species (ROS) by acting as a catalyst upon interacting with microbial cells. (5) Metal ions or NPs easily can enter microbial cells and can damage intracellular structures (e.g., via interactions with DNA and/or proteins) (Gordon et al., 2010; Grigor'eva et al., 2013). NPs can act as efflux pump inhibitors, and this activity might contribute to restoring the antimicrobial efficacy of antimicrobial agents, thereby reducing the resistance to antimicrobials (Ding et al., 2018). We mentioned that QS plays a critical role in biofilm formation and that QS inhibitors are considered promising antibiofilm alternatives. It has been identified that NPs can act as QS inhibitors to inhibit biofilm development (Masurkar et al., 2012; Radzig et al., 2013). (6) By using NPs as a carrier, compared with free loading of agents, antibacterial properties at a low dose against biofilm-derived planktonic cells and biofilms can be enhanced by improving the therapeutic index and the pharmacokinetic profile of the encapsulated antimicrobial drugs. NPs can reduce the toxicity and adverse side effects of these antimicrobial drugs (Ding et al., 2018; Fulaz et al., 2019). Silver NPs have been extensively researched for their antimicrobial properties, and we summarize the antibiofilm activities of silver NPs in Table 1.

**Table 1:** Anti-biofilm activity and application of silver APs.

**Pathogens**	**Effects**	**References**
*Pseudomonas aeruginosa*	Inhibiting biofilm formation	Sambhy et al., 2006; Besinis et al., 2014
*Staphylococcus epidermidis*	Inactivating enzymes	Gordon et al., 2010
*Pseudomonas aeruginosa, Escherichia coli, Staphylococcus aureus*	Inhibiting cell wall synthesis, protein synthesis, nucleic acid synthesis, QS	Masurkar et al., 2012; Radzig et al., 2013
*Salmonella typhimurium, Staphylococcus aureus*	Attaching to cell wall, damaging cell membrane, binding to DNA	Grigor'eva et al., 2013
*Candida albicans*	Inhibiting cell wall synthesis	Monteiro et al., 2013
*Pseudomonas aeruginosa*	Combined therapy with antibiotics, increasing penetration within biofilms	Habash et al., 2014
*Pseudomonas aeruginosa*	Influencing drug efflux as a carrier.	Ding et al., 2018
*A. baumannii, Escherichia coli, Staphylococcus aureus, Candida albicans*	Disrupting cell membrane, ROS generation, dissolving extracellular matrix	Paosen et al., 2019
*Staphylococcus aureus, Pseudomonas aeruginosa*	Modulating host immune response	Shi et al., 2019

Taken together, these advantages suggest that nanotechnology offers many promising opportunities to develop antimicrobial nanosystems to combat biofilms due to the unique mechanisms of the nanosystems, which are different from those of traditional antibiotics. Numerous materials have been produced and used in clinical trials, and several of them have been approved. Among these clinically approved NPs are lipid-, polymer- and protein-based NPs (Wolfram et al., 2015). Although most researchers have stated that NPs are safe for human tissues, certain categories of NPs have been reported to have cytotoxic effects (Zhao et al., 2008). Toxicological tests of NPs are limited, and further long-term studies for risk assessment of NPs are needed.

### Antimicrobial Peptides

Antimicrobial peptides (AMPs) are a series of compounds that are distributed widely in nature and are best known for their broad range of antimicrobial activity against viruses, bacteria, protozoa, and fungi. Natural AMPs are extracted from different kinds of live organisms, including vertebrates, invertebrates, plants, and bacteria. AMPs can also be produced by chemical synthesis. Compared with conventional antibiotics, both natural and synthetic AMPs play a broad range of antimicrobial roles without inducing the development of antibiotic resistance. Only a few AMPs can affect biofilms, and some of them show antibiofilm activity below the MIC, such as the human cathelicidin peptide LL-37 (Chennupati et al., 2009; Kai-Larsen et al., 2010). LL-37 presents very weak antiplanktonic cell activity, while its antibiofilm activity is much higher (Overhage et al., 2008). The mechanisms underlying the effect of AMPs against biofilms include (1) membrane-associated activity through pore formation and/or membrane disruption (Sochacki et al., 2011; Wang G. et al., 2014; Chen et al., 2019); (2) penetration into the cytoplasm of bacteria and suppression of cell wall, enzyme, or protein synthesis (Pinheiro et al., 2018); (3) degradation or destabilization of the extracellular matrix (Dean et al., 2011); and (4) prevention of cell attachment and promotion of existing cell dispersion in the early stages of biofilm development (Overhage et al., 2008; Kai-Larsen et al., 2010). Some AMPs can prevent or inhibit biofilm formation by inhibiting the QS of microorganisms (Overhage et al., 2008). The penetration of AMPs in biofilms is vital to their antibiofilm activity. AMPs can play an antibiofilm role by regulating host immune responses (Sol et al., 2013; Chen et al., 2019). Most AMPs are regarded as being toxic to only prokaryotic cells and not to host cells because of the difference in membrane structure (Ko et al., 2019). However, some studies have suggested that several factors, such as permeability, electric potential, fluidity, surface charge, hydrophobicity, and stability, may influence the effects of AMPs on the host membrane (Hoskin and Ramamoorthy, 2008). Some AMPs can electrostatically interact with the host cellular membrane and have been used in some anticancer research because of their antitumor activity (Zhou et al., 2018). Natural AMPs often have poor stability and proinflammatory effects; however, synthetic AMPs are designed to overcome these shortcomings of AMPs. However, compared to natural AMPs (Scott et al., 2002), synthetic AMPs show better instability by modifying the cleavage site of proteases, lowering toxicity, improving antimicrobial activity, and lowering production costs (Haisma et al., 2014; Pfalzgraff et al., 2018). Their potential toxicity and poor stability limit the use of AMPs in the clinical treatment of antibiofilm infections (Chennupati et al., 2009). Considering the promising advantages of AMPs, different combination strategies based on AMPs have been evaluated. AMPs combined with NPs by nanotechnology could penetrate the barrier of biofilms, and low doses of AMPs could be used to overcome their disadvantages and potential toxicities (Almaaytah et al., 2017).

Several AMPs with obvious antibacterial activities are secreted by human cells. Some agents could induce host cells to secrete AMPs against microbial infections, while some agents show a synergistic effect with AMPs secreted by host cells (Sechet et al., 2018). Some AMPs also show a synergistic effect combined with traditional antibiotics via the promotion of antibiotic uptake (Shurko et al., 2018). We list the antibiofilm activities of LL-37 and LL-37-derived peptides in Table 2.

**Table 2:** The anti-biofilm activities of LL-37 and LL-37-derived peptides.

**Pathogens**	**Effects**	***in vitro* or *in vivo***	**References**
*Pseudomonas aeruginosa*	Decreasing the attachment of cells, stimulating twitching motility, and influencing QS	*in vitro*	Overhage et al., 2008
*Pseudomonas aeruginosa*	Eradicating biofilms and decreasing bacterial counts with proinflammatory and ciliotoxic effects	*in vivo*	Chennupati et al., 2009
*Pseudomonas aeruginosa*	Inhibiting the attachment of cells and biofilm formation, degrading extracellular matrix	*in vivo*	Dean et al., 2011
*Escherichia coli*	Decreasing the attachment of cells, biofilm formation	*in vitro*	Kai-Larsen et al., 2010
*Escherichia coli*	Binding to the membrane, interfering with cell wall biogenesis	*in vitro*	Sochacki et al., 2011
*Aggregatibacter actinomycetemcomitans*	Regulating host immune responses	*in vitro*	Sol et al., 2013
*Staphylococcus aureus*	Disrupting bacterial membranes and binding DNA	*in vivo*	Wang G. et al., 2014
*Staphylococcus aureus*	Killing bacteria and inhibiting biofilm formation	*in vitro*	Haisma et al., 2014
*Staphylococcus aureus*	Synergic effect combined with other antibiotics	*in vitro*	Shurko et al., 2018
*Streptococcus mutans*	Disrupting cell membrane, inhibiting biofilm formation, inhibiting inflammation	*in vitro*	Chen et al., 2019

Some bacteriocins produced by almost all groups of bacteria present antibacterial activities, such as colicins and microcins. Colicins, produced by *E. coli*, and other colicin-like bacteriocins, produced by a range of Gram-negative bacteria, such as *P. aeruginosa*, kill bacteria closely related to the producing bacteria (Brown et al., 2012). Colicins can be divided into enzymatic colicins, which degrade nuclease function or inhibit cell wall synthesis, and pore-forming colicins, which depolarize the cytoplasmic membrane. Both of these mechanisms of action can lead to cell death (Rendueles et al., 2014). In addition, colicins and colicin-like bacteriocins are highly effective at killing target strains growing in the biofilm state (Brown et al., 2012). Microcins secreted by enterobacteria (mostly *E. coli*) also exert potent antibacterial activity against closely related species. They act by forming pores in the bacterial membrane, inhibiting aspartyl-tRNA synthetase, and inhibiting the DNA gyrase GyrB, resulting in DNA damage (Baquero et al., 2019). Microcins were used to fight a *P. aeruginosa* biofilm, and the killing activity of microcins against planktonic and mature biofilm cells was proven (Hwang et al., 2014). The use of these bacteriocins may become a new strategy for biofilm treatment. However, these compounds could also have functions involving interactions with eukaryotic host cells, inducing some degree of host DNA damage (Baquero et al., 2019), and these effects might limit their application in antibiofilm therapy.

These could be novel strategies to fight infections by regulating the host native immune system, and these treatments might simultaneously avoid the deleterious risks of an inflammatory response.

### Photodynamic Therapy

Photodynamic therapy (PDT), used to treat many diseases, utilizes a photosensitizer or photosensitizing agent followed by light of a specific wavelength. PDT was used as an antimicrobial strategy to inhibit biofilms formed by a broad spectrum of microorganisms by effectively damaging the cell membrane within the biofilm *in vitro* (Wood et al., 1999). Subsequently, antimicrobial PDT was found to nonspecifically attack microorganisms by generating cytotoxic ROS, which have strong oxidation ability and high reactivity, thus causing rapid lipid oxidation of the bacteria (Qi et al., 2019). It has been found that the antibiofilm activity of PDT is also associated with inhibition of the ability of microorganisms to adhere to surfaces, destroying biofilm structures, damaging some organelles, inducing virulence factor secretion, and inhibiting efflux capacity and QS (Arciola et al., 2011; Li et al., 2017; Tan et al., 2018; Hendiani et al., 2019; Mahdizade-Ari et al., 2019). Most antibiofilm studies of PDT have used this technique for dental plaque-related diseases and chronic wound infections (Zanin et al., 2005; Dilsiz et al., 2013; Mahmoudi et al., 2019); however, some studies have also used PDT to combat biofilm-related infections in ventilator-associated pneumonia and chronic rhinosinusitis (Krespi and Kizhner, 2011; Geralde et al., 2017). A majority of photosensitizers are poorly soluble in water and hydrophobic; however, with the application of NMs, this limitation might be overcome (Qi et al., 2019). PDT has emerged during the era of nanotechnology, and these combination strategies have been shown to have good effects (Khan et al., 2012). NMs can serve as photosensitizers to enhance photostability and the production of ROS, such as fullerenes, while NMs can also serve as nanocarriers for photosensitizers to increase stability, dispersity, and hydrophilicity, such as gold and silica-based NPs (Qi et al., 2019). Because of the continuous development of protocols and light sources, PDT has been used in long-term clinical trials. Most trials proved that PDT might become an antimicrobial therapy for biofilms, and no adverse effects of PDT were observed (Lopes et al., 2014; Alwaeli et al., 2015; Percival et al., 2015a; Tahmassebi et al., 2015). Despite promising results, conclusions from these studies should be carefully considered due to the limited number of included studies.

### Enzymes to Disperse Extracellular Polysaccharide Substances From Biofilms

The EPS of biofilms protects the cells inside biofilms from various antimicrobial agents and innate immunological responses. Enzymes targeting eDNA, extracellular polysaccharides, and proteins have been considered as strategies to eliminate biofilms (Kaplan et al., 2018). DNase I was identified as being effective in degrading eDNA *in vitro* and *in vivo* (Zhao et al., 2018), and it was used to impair biofilms, reduce microbial adhesion, and induce the dispersal of preexisting biofilms, especially early-stage biofilms. DNase I has been used to fight multiple biofilms, such as *C. albicans* (Martins et al., 2012), *S. enterica* (Wang H. et al., 2014), *Campylobacter jejuni* (Brown et al., 2015), *S. aureus* (Waryah et al., 2017), and *Burkholderia pseudomallei* (Pakkulnan et al., 2019) biofilms. Exopolysaccharides consisting of partially de-*N*-acetylated poly-β-d-(1,6)-*N*-acetyl-glucosamine (dPNAG) are the main structural components of Gram-positive and Gram-negative bacterial biofilm EPSs. Dispersin B, a new beta-*N*-acetylglucosaminidase from the oral pathogen *Aggregatibacter actinomycetemcomitans*, cleaves dPNAG and functions as a promising antibiofilm agent (Kaplan et al., 2018; Wang et al., 2019). Dispersin B was used as one of the components in multilayer coatings and exhibited high antibiofilm efficiency with high stability (Pavlukhina et al., 2012). Many other enzymes, such as proteinase K and lysozyme, have been proven to have promising antibiofilm activity (Eladawy et al., 2020). These biofilm-dispersing enzymes have better antimicrobial activities when administered in combination with antimicrobial agents (Darouiche et al., 2009; Rodríguez-López et al., 2017; Kim et al., 2019; Eladawy et al., 2020). Researchers have also tried different enzyme combinations (Karygianni et al., 2020) or dispersal-inducing enzymes combined with other new technologies, such as nanotechnology (Patel et al., 2019; Tasia et al., 2020), to improve antimicrobial biofilm activity and achieve good results. Thus, enzymatic treatment combined with conventional antimicrobial agents or other novel antibiofilm therapeutic agents provides us with another effective treatment strategy against biofilm-associated infections aimed at both bactericidal effectiveness and biofilm dispersal.

### Predatory Bacteria

*Bdellovibrio* and like organisms (BALOs) are a small group of bacteria that have the ability to target and prey on many Gram-negative bacterial species, such as *Klebsiella, Escherichia, Acinetobacter, Salmonella, Pseudomonas, Aeromonas, Vibrio, Shigella*, and *Yersinia* species (Dashiff et al., 2011; Duncan et al., 2019; Bratanis et al., 2020). The predation behavior of these organisms is a highly complex process: various prey are specifically recognized and killed, and the predator lives and replicates within the prey bacteria until all resources are exhausted (Lambert et al., 2009). Each predatory bacterium has its own prey spectrum. It was first reported in 2005 that *Bdellovibrio bacteriovorus*, the most well-studied predatory bacterium, could attack *E. coli* and *Pseudomonas fluorescens* biofilms as well as planktonic bacteria (Kadouri and O'Toole, 2005). BALOs can inhibit biofilm formation and reduce preexisting biofilms of prey bacteria. Gram-positive bacterial biofilms induce an intracellular transcriptome response in *B. bacteriovorus*, leading to the secretion of several proteases, hydrolases, and nucleases, which is associated with the degradative effect of BALOs on Gram-positive bacterial biofilms (Im et al., 2018; Bratanis et al., 2020). *in vivo* animal models have demonstrated that predatory bacteria are nontoxic and nonimmunogenic in rodents (Russo et al., 2015), while *in vitro* predatory bacteria are nonpathogenic and nontoxic to several kinds of human cell lines (Gupta et al., 2016). Despite the limitations of predatory bacteria, such as their potentially negative effect on the natural microbiota of the body and their incomplete predation of prey bacteria (Shatzkes et al., 2017), these bacteria are still regarded as “living antibiotics,” and researchers hope that these bacteria can serve alternatives to traditional antibiotics.

## Conclusions

Biofilm-related infections remain a serious concern in clinical services. The high resistance of biofilms to current antibiotic therapies seems to be a major challenge in this field. Biofilm eradication, whether in medicine or industry, is remarkably difficult. Antibiotic therapy alone often fails to eradicate microbial biofilms. Accompanied by a deeper understanding of the mechanisms of biofilm resistance, many developments, such as AMPs and nanotechnology, have been made in recent years and have been identified as effective and promising. Some of these strategies have antibiofilm activities against multiple targets. By combining these promising agents with antibiotics, the eradication of biofilms may be possible in the future. Nevertheless, to develop safe, effective, practical, and economically viable strategies against biofilm infections, further careful efforts are still needed in the field, including well-designed clinical trials.

## Author Contributions

YW conceived the general idea and provided critical revision and final approval of the manuscript. KZ, XL, and CY conducted the literature study and wrote the draft manuscript. All authors contributed to the article and approved the submitted version.

## Conflict of Interest

The authors declare that the research was conducted in the absence of any commercial or financial relationships that could be construed as a potential conflict of interest.
